# The complete chloroplast genome of Korean *Gastrodia elata* Blume

**DOI:** 10.1080/23802359.2020.1721346

**Published:** 2020-02-03

**Authors:** Min-Jeong Kang, Sang-Chul Kim, Hyo-Ryeon Lee, Sang-A Lee, Jei-Wan Lee, Tae-Dong Kim, Eung-Jun Park

**Affiliations:** Department of Forest Bio Resources, National Institute of Forest Science, Suwon, Republic of Korea

**Keywords:** *Gastrodia elata* Blume, chloroplast genome, sequence variants

## Abstract

The completed chloroplast genome of *Gastrodia elata* Blume (*G. elata*) from Korea was determined in this study. The cpDNA is 35,230 bp in length and lacked the large and small single copy (LSC and SSC) regions, due to the lost inverted repeat (IR). The overall AT content is 73.30%, and the cpDNA contains 20 protein-coding genes, 5 tRNA genes, and 3 rRNA genes. Remarkably, the Korean *G. elata* cp genome was 74 bp smaller than that of the Chinese *G. elata*. It revealed substantial sequence variants 495 SNPs and 75 InDels between the two *G. elata* genomes.

*Gastrodia elata* Blume (*G. elata*), as an obligate mycoheterotrophy, relies on fungi for seed germination and nutrient absorption due to lack of chlorophyll. *G. elata* is a traditional medicinal orchid, mainly used for treatment of convulsion ischemia, dementia, tremors, and vertigo. *G. elata* has been widely cultivated in the mountainous area of the Republic of Korea, China, and Japan. To understand the genetic history and relationship, we characterized the completed chloroplast (cp) genome sequence of *G. elata* grown in Korea for species identification and phylogenetic analysis.

Plant material of *G. elata* was collected in Geochang (35°4′N, 127°54′E), Republic of Korea. Genomic DNA was extracted from fresh stem tissue using the modified CTAB method and stored in the Department of Bio resources, National Institute of Forest Science (No. 20171205; Suwon, Korea). High-quality genomic DNA was sheared, and the library preparation and sequencing were performed by PacBio sequencing. The filtered sequences were assembled using the plastome of Chinese *G. elata* as a reference sequence (MF163256; Yuan et al. 2018). The sequenced fragments were assembled using Geneious R10 (Kearse et al. 2012) and annotation was performed using both DOGMA and BLAST searches. The tRNA genes identified using Geneious were validated using the web-based tool tRNAScan-SE (Schattner et al. 2005) with default settings. The annotated genome sequence was deposited into GenBank under the accession number MN026874.

The circular genome is 35,230 bp in length with an overall AT content of 73.30% and lacks the large and small single copy (LSC and SSC) regions due to the lost inverted repeat (IR). The cp genome contains 20 protein-coding genes, 3 rRNA genes, and 5 tRNA genes. Remarkably, the Korean *G. elata* cp genome is 74 bp smaller than that of the Chinese *G. elata* (35,304 bp, 73.23% of the average AT content). A total of 495 SNPs and 75 InDels were retained between the two genomes. Of these, 405 SNPs (81.8%) were distributed in the genic region, 90 SNPs (18.2%) in the intergenic region. 39 InDels occurred in the protein-coding region, most of which (87%) were less than 10 bp in length. Insertion occurred in the seven protein-coding genes (*clpP*, *matK*, *rps12*, *rpl2*, *rpl16*, *ycf,* and *ycf2*), while deletion was observed in the four coding genes (*clpP*, *rpl16*, *rps12*, and *ycf2*).

The whole cp genome sequence of Korean *G. elata* was aligned together with 12 completed plastomes of the Orchidaceae subfamily (Chao et al. 2016). Neighbor-joining tree and NJ bootstrap searches were performed using MEGA X (Kumar et al. 2018). The neighbor-joining method was run using rapid bootstrap analysis with a random starting tree and 1000 bootstrap replicates (Felsenstein 1985). A total of 13 plastomes were clustered together, Korean *G. elata* was closely related to Chinese *G. elata* (Figure 1).

**Figure 1.**
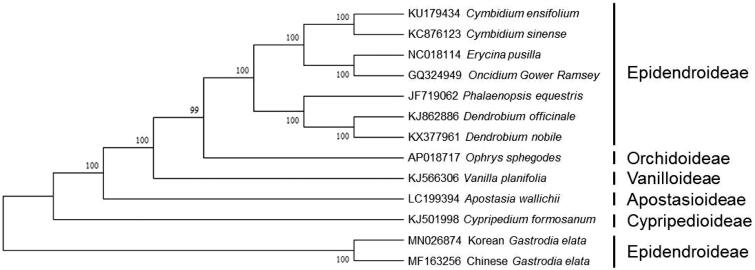
Phylogenetic analysis of *Gastrodia elata* with 12 members of the Orchidaceae subfamily based on the completed chloroplast genome sequences. The tree was constructed neighbor-joining method and bootstrap values from 1000 replicates. GenBank accession numbers are shown in the left panel.

Our study has shown the existence of substantial sequence variation in the plastome of *G. elata* corresponding to different geographic locations. Consequently, information obtained from the present study can be used for developing molecular markers and will provide a useful resource for population studies.
